# Role of miRNAs in vascular development

**DOI:** 10.1016/j.ncrna.2022.09.010

**Published:** 2022-09-29

**Authors:** Albert Sufianov, Sema Begliarzade, Valentin Kudriashov, Radmila Nafikova, Tatiana Ilyasova, Yanchao Liang

**Affiliations:** a Educational and Scientific Institute of Neurosurgery, Рeoples’ Friendship University of Russia (RUDN University), Moscow, Russia; b Department of Neurosurgery, Sechenov First Moscow State Medical University (Sechenov University), Moscow, Russia; c Republican Clinical Perinatal Center, Ufa, Republic of Bashkortostan, 450106, Russia; d Gastric Cancer Center, West China Hospital of Sichuan University, China; e Republican Children's Clinical Hospital, Ufa, Republic of Bashkortostan, 450106, Russia; f Department of Internal Diseases, Bashkir State Medical University, Ufa, Republic of Bashkortostan, 450008, Russia; g Department of Neurosurgery, The First Affiliated Hospital of Harbin Medical University, Harbin, 150001, China

**Keywords:** Vascular development, Regulation, Endothelial cells, Noncoding RNAs, microRNAs

## Abstract

The development of the vertebrate vascular system is an extremely important and complex process. The circulatory system is the first organ system to develop during embryogenesis. The development of the vasculature into highly branched canals must occur clearly in many places in order to supply oxygen and nutrients to the rapidly developing embryo. This process is mediated by a coordinated response of vascular endothelial and parietal cells to heterogeneous angiogenic signals provided by tissues and organs. MicroRNAs regulate gene expression at the transcriptional and post-transcriptional levels and participate in many important physiological and pathological processes. MicroRNAs mainly play an important role in the developmental regulation of vascular smooth muscle cells and vascular endothelial cells. This article summarizes the research progress of microRNAs in vascular development in recent years, focusing on the regulatory mechanism of miR-126 and miR-17/92 families in vascular endothelial cells, as well as the miR-143/145 family, miR-21 in vascular smooth muscle cell's regulation. The research prospects of the role of microRNAs in vascular development are also presented in this article.

## 1 Introduction

In the 1950s, scientists used light microscopy to discover that invertebrates lack endothelial cells in the blood circulation system, so invertebrates cannot undergo a series of evolutions like vertebrates to form a closed vascular system [1]. The appearance of closed vascular system in vertebrates contributes to the growth and development of vertebrates, and is also an important link in the process of biological evolution. The vascular system provides oxygen transport and various nutrient supply for the growth, development and survival of individual organisms. During embryonic development, disturbances in the development of the vascular system may lead to embryonic death or individual developmental disability. In higher vertebrates, vascular development is a very complex process. There are two vascular systems in higher vertebrates, the blood vascular system and the lymphatic vascular system [2]. There are certain differences between the lymphatic vascular system and the blood vascular system: blood vessels are a ring-shaped system, while the lymphatic vascular system is a linear system; capillaries are surrounded by vascular endothelial cells, and the endothelial cells that make up capillary lymph have connections between them similar to imbricate, where one cell edge overlaps the other [3]. The two are related to each other in the functions of tissue fluid reabsorption [2]. The existence of the blood vascular system and the lymphatic vascular system and the close connection between the two are essential in higher vertebrates [4]. MicroRNAs are a class of single-stranded non-coding small RNAs with a length of 21–23 nt, which function by recognizing the 3′ untranslated region sequence of the target mRNA and binding to its target, and inhibiting the translation level of its target after transcription [[5], [6], [7], [8], [9], [10]]. At present, there have been many reports on the regulation of microRNAs in vascular development. This article mainly reviews the research progress of microRNAs in the regulation of vascular development.

## 2 microRNAs in vascular development

MicroRNAs widely exist in various animal and plant genomes, maintain high homology and conservation during species evolution, and have strict expression specificity and timing in species [11]. In 1993, Lee et al. discovered the first microRNA in *Caenorhabditis elegans*, which is antisense complementary to the temporal gene lin-4 that regulates larval embryo development [12]. So far, thousands of microRNAs have been found in Drosophila, *C. elegans*, mice, and humans. Studies have shown that microRNAs can not only play a regulatory role at the post-transcriptional mRNA level, but also mediate many key physiological and pathological processes at the transcriptional level, including cell proliferation, cell fate determination, cell differentiation, cell metabolism, cell apoptosis, etc., so it's an important class of non-coding RNAs [13].

## 3 microRNAs affecting vascular endothelial cells

**Fig. 1:**
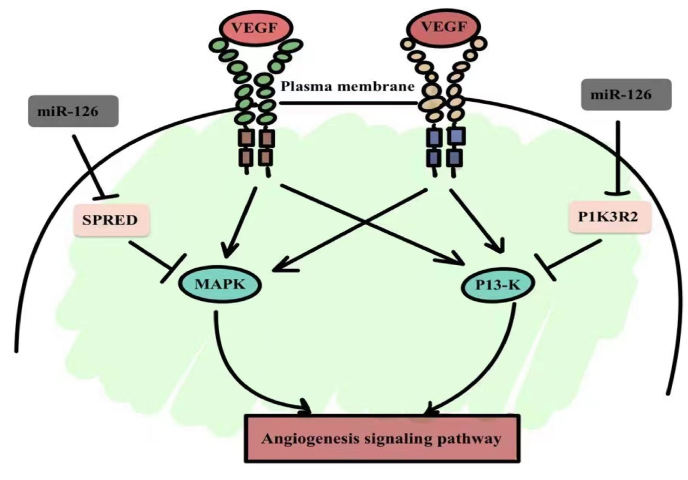
The regulation of the angiogenesis of the miR-126 through the signaling pathways. VEGF, vascular endothelial growth factor; PI3K, hosphoinositide 3-kinase pathway; MAPK, mitogen-activated protein kinase pathway; PI3KR2, phosphoinositide 3-kinase, regulatory subunit 2; SPRED1, Sprouty-related, EVH1 domain-containing 1.

The miR-17/92 family includes six microRNAs: 17, 18a, 19a, 20a, 19b-1 and 92a-1 [14]. Studies have shown that lack of this microRNA family leads to postnatal death in mice due to cardiac septal defect [15,73]. This family is highly expressed in endothelial cells and inhibits angiogenesis by hindering endothelial cell motility [16]. The miR-17/92 family regulates the expression of proto-oncogenes, and this family of microRNAs downregulates the expression of anti-angiogenic molecules, thrombospondin and connective tissue growth factor [17]. There are many studies on individual microRNAs in this family, for example, inhibiting the expression of miR-92a can enhance the growth of blood vessels during ischemic injury or myocardial infarction [18]. In vitro experiments, overexpression of miR-92a can inhibit the formation of vascular sprouting and vascular network; in animal experiments, overexpression of miR-92a inhibits the expression of angiogenic growth factors [19]. Other members of the miRNA-17/92 family, including miR-17 and miR-20a, also inhibit angiogenesis [20]. Studies have shown that miR-126 is highly expressed in endothelial cells [21]. miR-126 inhibits PI3K and MAPK signaling pathways to promote angiogenesis and downregulate the expression of inflammatory adhesion molecules and vascular cell adhesion molecule 1 (VCAM1) (Fig. 1) [22]. miR-126 can inhibit the formation of atherosclerosis and increase the stability of platelets, can regulate the angiogenic signaling cascade, and can act as an anti-inflammatory mediator in endothelial cells to inhibit the inflammatory response [23]. These studies suggest that miR-126 can regulate angiogenesis in a range of pathological conditions, suggesting that it may play a role in future cancer therapy. When human umbilical vein endothelial cells are injured, overexpression of miR-126 significantly enhances the PI3K/Akt signaling pathway [24]. In mice, knockout of miR-126 resulted in rupture of blood vessels during embryonic stage, and it can increase the activity of pro-angiogenic factors in vascular injury in adult tissues, and promote the formation of new blood vessels [25]. Recent studies have shown that miR-221 can down-regulate vascular endothelial growth factor (VEGF) receptor signaling by regulating PI3K regulatory subunits [26]. The miR-221/222 family is the target gene of c-Kit and let-7f, which can promote angiogenesis through thrombospondin 1 [66]. Deep sequencing showed that miR-221 increased endothelial cells in zebrafish embryos [26]. Knockdown of miR-221 did not affect embryonic vascular development, but resulted in defects in angiogenesis and lymphatic vasculature, similar to the absence of vascular endothelial growth factor receptor 3 (VEGFR-3) [26]. Overexpression of miR-221 can cause changes in apical cell behavior, such as increased proliferation and migration [27].

MiR-378 promotes VEGF expression by competing with miR-125a for the same region of the VEGF 3′-UTR [28]. miR-378 promotes cell survival by targeting the genes Sufu and Fus-1, and regulates tumor angiogenesis by indirectly upregulating VEGF. Studies have shown that injection of miR-378-transfected cancer cells in mice produces larger blood vessels than injection of cancer cells alone; overexpression of miR-378 in tumor cells increases cell viability and reduces cell death and promotes tumor growth and angiogenesis [29]. The miR-23/27/24 families is widespread in vascularized tissues and endothelial cells. Silencing of miR-23 and miR-27 can inhibit the activation of VEGF on MAPK and PI3K/PKB signaling pathways, thereby inhibiting angiogenesis, and inhibiting choroidal angiogenesis after laser injury [30]. In the absence of intersegmental vessels in zebrafish embryos, downregulation of miR-27 induces venous remodeling and angiogenesis [31]. The loss-of-function phenotype of miR-27 can be compensated by repressing one of Sprouty or DLL4 genes, therefore, these two genes may be the main target genes of miR-27 in zebrafish vascular development [31].

In vascular endothelial cells, hypoxia can induce the expression of miR-210 [32]. miR-210 can promote the formation of capillary sprouting and reduce apoptosis by inhibiting Ephrin-A3 under hypoxic conditions [32]. Downregulation of miR-200b promotes angiogenesis in endothelial cells when the skin is damaged [33]. In endothelial cells, overexpression of miR-181b can inhibit the expression of nuclear factor-κB (NF-κB) responsive genes; in the stimulatory response of mouse vascular endothelial cells to pro-inflammatory factors, miR-181b decreased expression [34]. In addition to the microRNAs mentioned above, many microRNAs also affect the development of blood vessels. Table 1 lists some of the microRNAs that affect the development of blood vasculature, and lists their target genes and functions.

**Table 1:** Some microRNAs involved in the vascular development.

MiRNA	Subject	Method	Target gene	Function
Mir-126	Mouse	Knockout	F4C	Plays a role in the formation of new blood vessels [35]
	Mouse	Knockout	Egfl7	Plays an important role in embryonic blood vessel formation development [35]
	Mouse	Injection of miR-126	Cxcl12	Regulation of apoptotic body makes it have the function of anti-atherosclerosis [23]
	Human umbilical vein	Transfection of miR-126 endothelial cells	VCAM-1	Inhibits VCAM-1 and regulates vascular inflammation [36]
	Zebrafish	Knockdown	Flt4	Inhibits the development of lymphatic vessels in the face and torso [37]
	Mouse	Knockout	Flt4	Regulates the development of the lymphatic network [38]
	Zebrafish	Knockdown	Spred1	Enhances Spred1 activity [37]
	Human coronary endothelial cells	Knockdown	Spred1	Regulates Spredl expression [38]
	Endothelial progenitor cell	Overexpression	P13KR2	Regulates angiogenesis via targeting PI3KR2 [39]
	Zebrafish	Knockdown	Pak1	Regulates the expression of Pakl in endothelial cells and causing head hemorrhage in zebrafish [40]
Mir-126a	Zebrafish embryos	Knockdown	Cxcl12a	Regulates the formation of lymphatic vascular cavity [41]
Mir-92a	Vascular smooth muscle cells	Overexpression	MKK4, JNK1	Down-regulates MKK4 and JNK1 [42]
	Mouse	Knockout	Itga5	Damages the development of the neointima [43]
Mir-19a	Endothelial cell	Overexpression	Cyclin Dl	Inhibits endothelial cell proliferation via negatively regulating Cyclin Dl [44]
Mir-146a, mir-21	Human coronary smooth muscle cells	Overexpression	Notch2	Inhibits expression of Notch2 to regulate proliferation of smooth muscle cells [45]
Mir-146a	Human umbilical vein Endothelial cell	Overexpression	IRAKI	Down-regulates IRAKI [46]
	Vascular smooth muscle cells	Knockdown	NF-kB, KLF4	Regulates the proliferation and migration of vascular smooth muscle cells via targeting NF-kB and KLF4 [47,48]
Mir-155	Mouse	Knockout	MST2	Regulates vascular smooth muscle cells by down-regulating MST2 [49]
	Mouse	Knockout	TNF-a	Regulates vascular inflammatory response and proliferation of neointima [50]
	Mouse	Knockout	CCN1	Promotes angiogenesis [51]
Mir-10a	Mouse umbilical vein endothelial cells	Overexpression	BMP2	Reduces proliferation and migration of umbilical vein endothelial cells and the formation of lumen [52]
	Mouse smooth muscle cells	Transfection of miR-10a	HDAC4	Reduces smooth muscle cell differentiation [53]
	Human arterial endothelial cells	Knockdown	HOXAl	Inhibits the expression of HOXAl [54]
Mir-10a, mir-22	Endothelial progenitor cell	Overexpression	Hmga2	Inhibits Hmga2 expression [55]
Mir-100	Mouse	Silent expression	mTOR	Inhibits the formation of blood vessels [56]
Mir-296	Human umbilical vein endothelial cells	Overexpression	HGS	Regulates HGS and promotes angiogenesis [57]
Mir-378	NCI–H292 cells	Overexpression	HMOX1	Regulates HMOX1 and affects angiogenesis and growth of non-small cell lung cancer [58]
	Mouse	Injection of miR-378-transfected cancer cells	VEGF	Affects angiogenesis [28]
Mir-23/27	Endothelial cells	Overexpression	SEMA6A, SPROUTY	Inhibits the expression of SEMA6A and SPROUTY and promotes angiogenesis [30]
Mir-96	Vascular smooth muscle cells	Injection of *anti*-miR-96	BMP4	Regulates vascular smooth muscle cells via targeting BMP4 [59]
Mir-34a	Vascular smooth muscle cells	Overexpression	SIRTl	Down-regulates SIRTl and promotes senescence of vascular smooth muscle cells [60]
Mir-217	Vascular smooth muscle cells	Transfection of mimics	NMDAR	Inhibits proliferation of vascular smooth muscle cells [61]
	Human umbilical vein endothelial cells	Transfection of mimics	SIRTl	Inhibits SIRTl and regulates FoxOl resulting in angiogenesis damage and promotes endothelial cell senescence [62]
Mir-182	Zebrafish	Knockout	FoxOl	Regulates angiogenesis via targeting FoxOl [63]

## 4 microRNAs affecting vascular smooth muscle cells

**Fig. 2:**
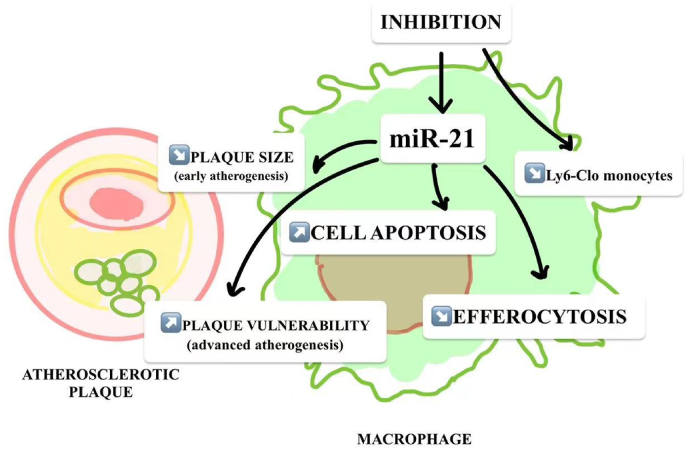
MiR-21 in regulating lipid metabolism, apoptosis, macrophage inflammation and efferocytosis during atherogenesis.

So far, many microRNAs related to vascular development have been reported, and many are related to vascular smooth muscle cells. For example, miR-21 has an important regulatory role in the proliferation and migration of vascular smooth muscle cells [64], and There are also higher levels of expression in endothelial cells [65]. Down-regulation of miR-21 expression increases apoptosis and inhibits the proliferation of adventitial fibroblasts and myofibroblasts [66]. miR-21 regulates vascular smooth muscle cell differentiation by affecting bone morphogenetic protein 4 (BMP4) and transforming growth factor β (TGF-β) signaling pathways [67]. miR-21 also regulates smooth muscle cells and endothelial cells, affecting vascular remodeling [15]. Studies have shown that the expression of miR-21 is increased in human atherosclerotic lesions (Fig. 2) [68].

In vitro experiments in serum-depleted conditions, human or mouse aortic smooth muscle cell differentiation reduces miR-21 expression [65]. In vitro experiments in mouse aortic smooth muscle cells and injured mouse carotid arteries, silencing of miR-21 will inhibit cell proliferation and increase apoptosis [65]. In mice, knockdown of miR-21 inhibits vascular remodeling in carotid injury [65]. Studies have shown that miR-21 has a higher expression level in various solid tumors than normal cells [69], so miR-21 can both promote cell proliferation and inhibit cell proliferation. Studies have shown that miR-146a can promote the proliferation of vascular smooth muscle cells in vitro and the intimal proliferation of angiogenesis in vivo [70]. Transfection of antisense miR-146a oligonucleotides into carotid balloon-injured mice significantly reduced neointimal hyperplasia [70], suggesting that miR-146a can promote vascular smooth muscle proliferation. At the same time, miR-146 can form a negative feedback loop to inhibit Toll-like receptor (TLR) signaling pathway, this negative feedback leads to endotoxin-induced tolerance to a certain extent, and can inhibit the production of inflammatory factors [71]. Studies have also shown that miR-147 and miR-155 also have similar functions [72]. Numerous studies have shown that vascular cell motility plays a crucial role in the development of various cancers and cardiovascular diseases, and the miR-143/145 family can regulate vascular cell motility [73]. In mice, knockdown of both miR-143 and miR-145 resulted in abnormal vascular tone and down-regulation of vascular smooth muscle cell-specific genes [74]. It has been shown that deletion of this family results in a reduced intimal proliferative response in vascular injury [74]. The miR-143/145 family is highly expressed in normal vascular smooth muscle cells, but the family is less expressed in acute and chronic vascular stress and human aortic aneurysms [75], and decreased expression in proliferating vascular smooth muscle cells [75,76]. In this family, miR-145 interacts with miR-143 to upregulate the expression of numerous target genes, including Kruppellike factor 4 (KLF4), Elk-1 (member of the ETS oncogene family) [76], vascular Angiotensin-converting enzyme (ACE) [77], serum response factor (SRF), and its co-activator, myocardin [74], It is shown that miR-145 can regulate the differentiation of human embryonic stem cells and the self-renewal of bone marrow stem cells. miR-143 can inhibit the migration of vascular smooth muscle cells through versican [78]. Platelet-derived growth factor induces miR-24 transcription, which induces a synthetic phenotype of vascular smooth muscle cells [79]. miR-24 is highly expressed in endothelial cells under stress conditions, such as oxidative stress. In mice, antisense expression of miR-24 enhanced angiogenesis and cardiac function [80].

Transfection of antisense miR-155 inhibitor in vascular smooth muscle cells can up-regulate the expression of endogenous angiotensin II type 1 receptor (AGTR1) [81]. miR-155 is expressed in smooth muscle cells, and its absence in atherogenesis reduces fat accumulation in macrophages [82]. The expression of miR-155 is up-regulated in mice and humans with atherosclerotic injury. Although up-regulation of miR-155 expression can promote atherosclerotic injury in humans, the circulating levels of miR-155 in patients with coronary artery disease are decreased [68]. Studies have shown that implantation of the bone marrow of LDLR-deficient mice with a high-fat diet into the bone marrow of miR-155-deficient mice will aggravate atherosclerotic lesions and inflammatory responses [82]. Down-regulation of miR-30b and miR-30c expression can lead to increased calcification of vascular smooth muscle cells [83]. Inhibition of miR-26a can accelerate the differentiation of vascular smooth muscle cells, and regulation of TGF-β signaling pathway by miR-26a may alter the phenotype of vascular smooth muscle [84]. During the proliferation of vascular smooth muscle and the growth of vascular wall neointima, the expression of miR-31 is significantly increased [85]; knockout of miR-31 can down-regulate serum and platelet-derived growth factors, thereby inducing vascular smooth muscle cell proliferation [85]. Overexpression of miR-208 can promote the proliferation of vascular smooth muscle cells, and can increase the regulatory effect of insulin on the proliferation of vascular smooth muscle cells [86]. In vascular smooth muscle cells, overexpression of miR-181a down-regulates the expression of angiotensin II (Ang II), up-regulates the expression of osteopontin (OPN), and enhances the adhesion of vascular smooth muscle cells to collagen [87]. In human aortic smooth muscle cells, overexpression of myocardin upregulates the expression of miR-1 and inhibits the proliferation of vascular smooth muscle cells [88].

## 5 Research on micrornas related to the development of lymphatic vasculature

MicroRNAs regulate not only the development of blood vasculature, but also the development of lymphatic vasculature. MiR-31 functions in early Xenopus embryonic lymphatic vasculature development [89]. Studies have shown that in lymphatic endothelial cells, knockdown of FAT4, which is a target gene of miR-31, enhances cell migration [90]. Studies have shown that both miR-31 and miR-181a are expressed in vascular endothelial cells during zebrafish embryonic lymphangiogenesis, and miR-31 or miR-181a regulates lymphangiogenesis by regulating the BMP2b/BMP2 signaling pathway [91]. In vitro experiments demonstrated that miR-184 inhibits corneal lymphangiogenesis, and overexpression of miR-184 reduces the migration of lymphatic endothelial cells and inhibits the formation of lymphatic endothelial cells [92]. In human lymphatic endothelial cells, overexpression of miR-27a reduces the formation and migration of lymphatic vessels, and the target gene of miR-27a is SMAD4, which negatively regulates the length of lymphatic vessels in the formation and migration of human lymphatic endothelial cells [93]. miR-206 inhibits tumor lymphangiogenesis in pancreatic ductal adenocarcinoma cells, thereby delaying tumor growth, which has certain significance in cancer therapy research [94]. Studies on the morphogenesis of lymphatic vessels in zebrafish have shown that miR-182 knockdown in zebrafish has defects in parachordal lymphatic vessels [63].

## 6 Conclusion

Vascular development is an extremely important and complex process, including the differentiation of endothelial cells, angiogenesis and angiogenesis, and the formation of lymphatic vasculature. It also involves many signaling pathways and transcription factors such as Notch and BMP. These regulatory factors control the differentiation and movement of endothelial cells, thereby regulating the development of blood vessels. Therefore, studying the mechanism of vascular development and its related signaling pathways has a certain role in promoting the evolution, growth and reproduction of animals. Many studies related to vascular development have shown that microRNAs play an important role in vascular development. MicroRNAs mainly inhibit vascular development or make vascular proliferation by regulating their target genes, and have vascular smooth muscle cells and vascular endothelial cells in the blood vascular system. Although many studies have revealed the regulatory mechanism of microRNAs in vascular development, many microRNAs with important functions in vascular development have not been discovered. Further exploration of the regulation of microRNAs on vascular development will help us to deeply understand the process of life, which is of great significance for the study of biological evolution.

## Author statement

Albert Sufianov and Sema Begliarzade: conceptualized and designed the study. All authors participated in the acquisition, analysis and interpretation of the data. Valentin Kudriashov, Radmila Nafikova: drafted the manuscript. Tatiana Ilyasova, Yanchao Liang: contributed to critical revisions of the manuscript. All authors agreed on the journal to which the article would be submitted, gave final approval for the version to be published, and agreed to be accountable for all aspects of the work.

## Funding

This study was supported by the Bashkir State Medical University Strategic Academic Leadership program (PRIORITY-2030).

## Declaration of competing interest

The authors declare that no conflicts of interest exist.
